# Biomarkers in Painful Symptomatic Knee OA Demonstrate That MRI Assessed Joint Damage and Type II Collagen Degradation Products Are Linked to Disease Progression

**DOI:** 10.3389/fnins.2019.01016

**Published:** 2019-10-15

**Authors:** Nidhi Sofat, Vivian Ejindu, Christine Heron, Abiola Harrison, Soraya Koushesh, Lena Assi, Anasuya Kuttapitiya, Guy S. Whitley, Franklyn A. Howe

**Affiliations:** ^1^Institute for Infection and Immunity, St George’s University of London, London, United Kingdom; ^2^St George’s University Hospitals NHS Foundation Trust, London, United Kingdom; ^3^Molecular and Clinical Sciences Research Institute, St George’s University of London, London, United Kingdom

**Keywords:** biomarkers, pain – etiology and drug therapy, sensitization, magnetic resonance imaging, arthritis

## Abstract

**Background:**

Osteoarthritis (OA) is the most prevalent arthritis worldwide, but the evolution of pain in relation to joint damage and biochemical markers are not well understood. We evaluated the relation between clinical pain measures and evoked pain in relation to structural damage and biochemical biomarkers in knee OA.

**Methods:**

A cross-sectional study in people with knee OA and healthy controls was conducted. A total of 130 participants with advanced OA requiring total knee replacement (TKR) (*n* = 78), mild OA having standard care (*n* = 42) and non-OA controls (*n* = 6), with four drop-outs were assessed. Pain scoring was performed by the Western Ontario and McMaster Universities OA Index (WOMAC_P) and the Visual Analog Scale (VAS). Pain sensitization was assessed by pain pressure thresholds (PPTs). Knee magnetic resonance imaging (MRI) assessed joint damage using the MRI Knee OA Score (MOAKS). Overall MOAKS scores were created for bone marrow lesions (BMLs), cartilage degradation (CD), and effusion/Hoffa synovitis (tSyn). Type II collagen cleavage products (CTX-II) were determined by ELISA.

**Results:**

The advanced OA group had a mean age of 68.9 ± 7.7 years and the mild group 63.1 ± 9.6. The advanced OA group had higher levels of pain, with mean WOMAC_P of 58.8 ± 21.7 compared with the mild OA group of 40.6 ± 26.0. All OA subjects had pain sensitization by PPT compared with controls (*p* < 0.05). WOMAC_P correlated with the total number of regions with cartilage damage (nCD) (*R* = 0.225, *p* = 0.033) and total number of BMLs (nBML) (*R* = 0.195, *p* = 0.065) using body mass index (BMI), age, and Hospital Anxiety and Depression Scale (HADS) as covariates. Levels of CTX-II correlated with tSyn (*R* = 0.313, *p* = 0.03), nBML (*R* = 0.252, *p* = 0.019), number of osteophytes (*R* = 0.33, *p* = 0.002), and nCD (*R* = 0.218, *p* = 0.042), using BMI and age as covariates. A multivariate analysis indicated that BMI and HADS were the most significant predictors of pain scores (*p* < 0.05).

**Conclusion:**

People with both mild and advanced OA show features of pain sensitization. We found that increasing MRI-detected joint damage was associated with higher levels of CTX-II, suggesting that increasing disease severity can be assessed by MRI and CTX-II biomarkers to evaluate OA disease progression.

## Introduction

Osteoarthritis (OA) is a disease of the whole joint and is the most prevalent arthritis worldwide (O’Neill et al., 2018). Recent work has shown how imaging tools such as magnetic resonance imaging (MRI) and ultrasound (US) can be used to observe widespread changes in the joint, including in articular cartilage, underlying bone, with the development of osteophytes (Ost), subchondral cysts, bone marrow lesions (BMLs), synovitis (Syn), and joint effusions (Roemer et al., 2016). The two most significant correlates of pain in OA are Syn and BMLs (Sofat et al., 2011).

Recent cohort studies, especially of knee OA, have shown how painful OA is more likely to be associated with Syn measured by US or MRI and regions of established cartilage loss with underlying subchondral bony changes and/or BML, which are associated with more symptomatic disease (Crema et al., 2013; Deveza et al., 2017; Roemer et al., 2018). In addition, the biopsychosocial model of pain may explain why people with relatively modest levels of structural damage in OA may report high levels of pain and functional impairment (Arendt-Nielsen, 2017). Although there is evidence to suggest that imaging and biochemical markers are related to OA structural damage and progression in several studies (Hunter et al., 2011b; Mobasheri et al., 2017), information from clinical measures (including pain sensitization), biochemical, and imaging dimensions have rarely been combined in single reported analyses, so there remains a lack of clarity in managing pain and functional impairment in OA using the biomarkers reported (Dam et al., 2009; Gregori et al., 2018). In the pursuit of new disease modifying OA drugs (DMOADS), of which several are under investigation, robust biomarkers of disease severity and progression are urgently needed for evaluation with clinical outcomes.

Our group and others have previously shown that degradation products of type II collagen and MRI of the knee represent biomarkers that show promise as distinctive measures of damage in painful OA (Hunter et al., 2011a; Kuttapitiya et al., 2017). In this paper, we demonstrate how pain sensitization is a feature of mild and advanced OA. We also report how anxiety and depression factors may influence pain perception in knee OA. To our knowledge, this is the first study evaluating data on knee OA for pain sensitization, anxiety and depression scores, MRI changes measured by the MRI Knee OA Score (MOAKS), and the biochemical marker CTX-II in a cross-sectional study design from a UK dataset.

## Materials and Methods

All procedures were carried out after Ethical approval was granted (Health Research Authority approval number 12/LO/1970). In order to compare the potential use of biomarkers for pain, MRI, and biochemical changes, we designed a case–control study comparing subjects with different stages of OA pathology. There were three groups in our study: advanced OA, mild OA, and healthy controls (who did not have arthritis). For the “advanced OA” group, participants attending the South West London Elective Orthopaedic Centre were recruited at the time of assessment for total knee replacement (TKR). Study criteria for recruitment were as previously described (Kuttapitiya et al., 2017). The power calculation was made to detect differences in pain outcome in the “mild OA” and “advanced OA” groups. The control group was primarily recruited for absence of pain comparisons and biomarker levels. For the WOMAC Pain score outcome, we aimed to detect a mean difference of 15 points between the “advanced OA” and “mild OA” groups with a standard deviation of 26. With 48 subjects per group (“mild OA” and “advanced OA,” respectively), 80% power with a 0.05 significance level (two sided) is achieved.

The “mild OA” group had knee arthritis under medical management and physiotherapy. The study sample size estimation was to recruit at least 100 subjects, and we were able to recruit 126 subjects whose results could be evaluated.

### Study Consent, Control Participants, and Inclusion/Exclusion Criteria

For subjects with OA, all participants were aged 35–90 years, had pain, and fulfilled American College of Rheumatology (ACR) criteria for knee OA (Kuttapitiya et al., 2017). The reason for the broad age of recruitment reflected the usual age of presentation for mild OA (younger, aged 35 years or over) and advanced OA (older, aged 50 years or over). Subjects were consented by a research associate independent of the treating physicians, for knee MRI of the target joint and for waste joint tissue collection at TKR. All participants with knee OA underwent baseline knee radiography to confirm knee OA as part of their routine clinical management and subjects with a Kellgren–Lawrence grade of greater than or equal to two were recruited. The “mild OA” group was defined as participants who had painful knee OA and were being managed with analgesics, e.g., opioids, non-steroidal anti-inflammatory drugs (NSAIDs), and/or physical therapies, but were not deemed by their physician to require joint replacement surgery. Participants with “advanced OA” knee were those who had already received full medical management, but still experienced pain and deemed suitable by their clinician candidates for TKRs. Exclusion criteria for the “mild” and “advanced” knee OA groups were: other rheumatologic diagnoses, e.g., rheumatoid arthritis, systemic lupus erythematosus, pregnancy, regular use of bisphosphonates, corticosteroids, hormone replacement surgery in the last 6 months, history of clinically diagnosed depression, anxiety, or other recent surgery.

Participants without knee OA were recruited as control subjects. Inclusion criteria for healthy controls were: age 35–90 years, male or female, no previous history of knee injury, fractures, OA, or inflammatory arthritis. Exclusion criteria for the healthy control group were: current use of analgesics, e.g., opioids or NSAIDs, other rheumatologic diagnoses, e.g., rheumatoid arthritis, systemic lupus erythematosus, pregnancy, regular use of bisphosphonates, corticosteroids, hormone replacement surgery in the last 6 months, history of clinically diagnosed depression, anxiety, or other recent surgery. Healthy subjects volunteered of their own free will and were screened for suitability.

### Clinical Scores and Pain Assessments

Clinical scores were collected for participants with mild OA, advanced OA, and healthy controls. The pain subscales for the Western Ontario and McMaster Universities OA Index (WOMAC) were recorded for each participant at enrolment into the study. Participants were asked to score based on their symptoms in the last 48 h. The WOMAC is a well-validated pain scoring system and is one of the most widely used pain assessment tools in OA clinical studies (Bellamy et al., 2015). We used the WOMACVA3.1 questionnaire, which comprises of questions for the pain (5 questions), stiffness (2 questions), and function (17 questions) subscales. Since this report focuses on structural and biochemical correlations with pain, only results for the WOMAC Pain subscale (WOMAC_P) are reported. Data for WOMAC stiffness and function scores are reported elsewhere (Kuttapitiya et al., 2017). In addition to WOMAC_P, we collected data for all participants for the Visual Analog Scale (VAS) for pain on a rating scale of 0–10, since VAS pain is a pain outcome measure recommended by IMMPACT in clinical pain studies (Dworkin et al., 2005). Data were collected for body mass index (BMI), defined as weight/height^2^, since obesity is a recognized risk factor for OA development. It is also recognized that anxiety and depression can influence and impact on pain reporting (O’Neill et al., 2018), therefore data using the Hospital Anxiety and Depression Scale (HADS) (Bjelland et al., 2002) were also collected. The HADS scale consists of 14 questions assessing depression and anxiety. The questionnaire is scored out of 21, with a score of 0–7 being normal, a score of 8–10 suggesting borderline features of anxiety or depression, and a score of between 11 and 21 suggesting features of anxiety/depression requiring assessment and treatment. Quantitative Sensory Testing (QST) by pain pressure thresholds (PPT) were determined using a Somedic Algometer as previously described (Kuttapitiya et al., 2017).

### Quantitative Sensory Testing by Pain Pressure Thresholds

Briefly, PPT was performed pre-operatively before knee replacement. The somatosensory responses were assessed via pain pressure algometry. Increasing pressure was applied at a predetermined rate; known as the “slope” (10 kPA/s), to a test site using a handheld algometer (Algometer Type II, SBMEDIC Electronics, Solna, Sweden). In this way the subject’s PPT level of sensitivity to pain and function of non-myelinated C-fibers could be determined. Test sites were the target knee and contralateral knee along with three distal points. The knee was sub-divided into five areas (patella, lateral femur, medial femur, medial tibia, and lateral tibia) and the distal points included the target lateral malleolus and the right and left radius. Three readings were taken at each test point: the first being a test reading. Average PPT scores are reported for target and contralateral knee (PPT_TK, PPT_CK) across the five sites, the average for left and right radius (PPT_R), and for the individual sites of patella of target and contralateral knee (PPT_TK_Pa and PPT_CK_Pa) and malleolus (PPT_M).

### Biochemical Markers

Biochemical markers were obtained in a subset of patients and healthy controls. CTX-II levels were determined using ELISA to detect C-terminal telopeptides of type II collagen cleavage products from urine samples with creatinine levels for normalization as previously described (Kuttapitiya et al., 2017). Urine samples were acquired prior to MRI scans.

### Magnetic Resonance Imaging

**FIGURE 1:**
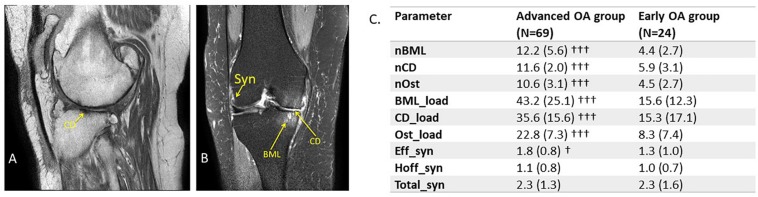
Measurement of MOAKS scores in subjects with knee OA. **(A)** Representative MRI scans acquired at 3T with an eight channel knee coil for patients with advanced OA. **(A)** Sagittal T1-weighted image with TE 15 ms, TR 600 ms, Subject 38, average cartilage damage score (CD_load) = 94. **(B)** Coronal intermediate weighted image with TE 30 m, TR 5000 ms, fat saturation, 0.35 mm in plane resolution, 3 mm slice thickness, 0.25 mm slice gap, SENSE factor 1.4. Subject 58, total synovitis score (total_Syn) = 3 (range 0–6), whole knee scores for BML (BML_load) = 44 (range 0–125), CD (CD_load) = 46 (range 1–94), and osteophytes (Ost_load) = 19 (range 0–38). Ranges given are the minima and maxima over the whole patient data set. **(C)** Comparison of MOAKS’ defined structural damage in knee OA in advanced versus mild OA. Significant differences by Mann–Whitney *U*-test were: ^†^*p* < 0.05, ^†††^*p* < 0.001 between advanced and early OA groups. Syn, synovitis; BML, bone marrow lesion; CD, cartilage degradation; Ost, osteophyte.

Magnetic resonance imaging of the target knee was acquired from OA patients within 6 weeks prior to TKR to enable visualization of structural changes, including BML, Syn, and cartilage damage (CD). Data were obtained using a 3T scanner with an eight-channel knee coil and included sagittal T1-weighted (TE 15 ms, TR 600 ms) images for delineating knee anatomical structures and fat-suppressed intermediate-weighted (TE 30 ms, TR 5000 ms) images in sagittal, coronal, and axial image planes for visualization of Syn and BMLs (Figure 1). The multimodal MRI was obtained within 30 min using protocols that complied with scanner safety procedures with adherence to any contra-indications for MRI scanning. Radiographic changes for structural damage features of CD, BMLs, Hoffa and effusion Syn, and osteophytes (Ost) were evaluated using the validated MOAKS (Hunter et al., 2011a). MOAKS evaluation was performed by two consultant radiologists (VE and CH) who were blinded to the patient’s clinical outcome and group allocation. Consensus scores were then reached for all of the individual scores for each anatomical region assessed. The MOAKS variables were calculated for MRI of the target knee for TKR in subjects with advanced OA and in the most affected knee for subjects with mild OA who were managed by usual care with analgesia and physical therapies.

Magnetic resonance imaging Knee OA Score includes evaluation over 15 anatomical regions for BMLs, 14 regions for CD and Ost, and two regions for Syn (Hunter et al., 2011a). The individual MOAKS values were combined to provide global measures of damage to enable creation of continuous variables that could be used in correlation analysis with clinical and biomarker parameters. Hoffa and effusion Syn score were combined to create a total Syn score (Total_Syn). The number of BMLs were summed over all anatomical regions to give the total number over the whole knee (nBML). A similar process was performed for the number of regions identified with CD (nCD) and for the number of osteophytes (nOst). For BMLs a lesion load score was evaluated as the product of number of lesions, lesion size score, and the % area score of lesion size, for each of the individual regions evaluated in MOAKS. These individual scores were then summed to give a whole knee lesion load (BML_load). A similar process was performed for CD and Ost by summing the product of number of lesions with lesion size scores over these same anatomical regions to give load scores (CD_load, Ost_load) for the whole knee.

### Statistical Analysis

Data were analyzed using IBM SPSS Statistics 25 and statistical significance was considered at a threshold of *p* < 0.05. We make no explicit multiplicity corrections for this exploratory data analysis. The Mann–Whitney *U*-test was used for group comparisons and bivariate analysis using a direct Pearson correlation and with suitable covariates and was used to assess relationships between pairs of all clinical, MRI, and fluid biomarker parameters.

A multivariate analysis was performed using the Automatic Linear Modeling module in SPSS, which is a regression technique that determines a linear combination of a defined set of parameters that best describes the variability of a specific dependent variable. The dependent variables assessed were the VAS and WOMAC pain scores, the PPT at the patella, and the urinary biomarker CTX-II. We assessed how these parameters related to the structural damage observed by MRI as defined by the MOAKS load scores for BML, CD, Ost, and the total Syn. BMI and Age were also included in the model and for the pain scores HADS was additionally included. Data sets were preselected to include only those for which all parameters were available for each analysis. The adjusted *r*-squared was used for the optimization parameter and all parameters were used in the “best-fit” option. We report the significant coefficients of the linear modal and the overall accuracy of the fit.

For MRI measurements recorded for the two scorers (VE and CH), inter-rater reliability and correlation were calculated using Kendall’s tau-*b*, which is suitable for ordinal data and Spearman correlation. We assessed the reliability across the BML, CD, and Ost MOAKS scores at the level of individual anatomical regions, as well for the total number of lesional regions per patient for BML, Ost, and CD. In accordance with reliability criteria set by Landis and Koch (1977), 0.61–0.8 suggests very good reliability, while 0.81–1.0 suggests near perfect agreement.

Data from all investigations were not available for all participants, hence statistical calculations were performed across the maximum possible number of patients within each analysis.

## Results

Our cross-sectional study identified a total of 120 participants at distinct stages of management by standard care for their OA. Subjects were evaluated in two OA groups: the “advanced OA” group (*n* = 78) who underwent TKR for knee OA, and a “mild OA” group who were being treated with medical management (*n* = 42). All subjects were prescribed NSAIDs or opioid analgesics for their arthritic pain. Clinical information and pain scores were obtained from all 120 participants, QST measurements from *n* = 118, wet biomarkers quantified from *n* = 112, and MRI performed on *n* = 93 OA patients. In a third “healthy control” group (*n* = 6) who had no evidence of arthritis we obtained pain scores, wet biomarker levels, and PPT measurements for indicative comparisons with the participants with OA. There were four drop-outs in the study where data could not be assessed.

Study demographics (Table 1) showed significantly higher BMI, WOMAC Pain (WOMAC_P), and VAS, with significantly lower patella PPT (Table 2) in participants with OA (advanced and mild groups) compared with healthy non-OA controls. WOMAC_P and VAS were higher in participants with advanced OA compared with mild OA. A Chi-squared comparison did not show any significant differences in gender in each of the groups (*p* = 0.16). The comparison to healthy volunteers is considered of importance due to the significant clinical differences (Table 1) between these groups. The healthy controls had no evidence of pain or OA clinically. Although the healthy control group was significantly younger than the OA groups, they were recruited for age and lack of OA or pain at the age when OA often first presents (aged 45 years or over). Since the age range for recruitment into the study included subjects from 35 to 90 years, due to the wide age range for OA presentation, the differences in age for our three groups are reflected by this. Subjects with advanced OA were the oldest (mean age 69 years) and had the highest levels of pain and MRI-reported structural damage, with the mild OA group being slightly younger with a mean age of 64 years. Although the healthy control group were significantly younger than the OA groups, with a mean age of 45 years, they were recruited for meeting criteria for age and lack of OA or pain at the age when OA often first presents (aged 35 years or over).

**TABLE 1:** Study demographics.

	**Advanced OA group**	**Mild OA group**	**Healthy volunteers**
**Parameter**	**(*n* = 78)**	**(*n* = 42)**	**(*n* = 6)**
Age (year) Age range (year)	68.9(7.7)^††^51 - 88	64.1(9.6)47−85	45.0(5.6)^∗∗∗^36−51
Female, *N* (%)	50(64)	30(71)	6(100)
Body mass index (kg m^–2^)	32.3(5.6)^††^	29.2(4.7)	23.3(3.6)^∗∗∗^
WOMAC pain (0–100)	58.8(21.7)^†††^	40.6(26.0)	0.6(1.4)^∗∗∗^
VAS pain (0–10)	5.8(2.3)^†††^	3.7(2.8)	0(0)^∗∗∗^
HADS (0–20)	12.5(7.1)	13.3(7.4)	7.5(5.8)
PPT (kPa)	394(223)	423(227)	690(192)^∗∗^
Clinical management	Total knee replacement	Medical management	None

*Study demographics are shown as mean and standard deviation in parentheses. WOMAC, Western Ontario and McMaster Universities Osteoarthritis Index; VAS, Visual Analog Scale; HADS, Hospital Anxiety and Depression Scale; PPT, pain pressure threshold. Significant differences with a Mann–Whitney *U*-test were: ^∗∗^*p* < 0.01, ^∗∗∗^*p* < 0.001 between healthy controls and all OA subjects. ^††^*p* < 0.01, ^†††^*p* < 0.001 between advanced and mild OA groups. A Chi-squared test was performed to evaluate any female proportion comparisons. This showed a Chi-square value of 3.60, degrees of freedom 2, with a *p*-value of 0.16.*

**TABLE 2:** Quantitative sensory testing (QST) by pain pressure thresholds (PPTs) in knee OA.

	**Advanced OA group**	**Early OA group**	**Healthy volunteers**
**Parameter**	**(*N* = 76)**	**(*N* = 42)**	**(*N* = 6**
PPT_TK_Pa	392(225)	423(227)	690(192)^∗∗^
PPT_CK_Pa	427(327)	453(210)	628(62)^∗^
PPT_TK	366(214)	339(155)	454(115)
PPT_CK	376(179)	367(153)	452(99)
PPT_TK_M	345(183)	359(164)	1020(231)^∗∗∗^
PPT_R	381(186)	374(143)	500(63)

*PPTs are shown as mean and standard deviation in parentheses in kPa. Significant differences with a Mann–Whitney *U*-test were: ^∗^*p* < 0.05, ^∗∗^*p* < 0.01, ^∗∗∗^*p* < 0.001 between healthy controls and all OA subjects. There were no significant differences between advanced and early OA groups (*p* > 0.3). PPT, pain pressure threshold; PPT_TK_Pa, PPT for the target knee at the patella; PPT_CK_Pa, PPT at the contralateral patella; PPT_TK, PPT at for the whole knee; PPT_CK, PPT for the contralateral knee; PPT_TK_M, PPT for the target knee side at the malleolus; PPT_R, PPT at the right radius.*

Evoked pain assessed by QST showed that PPT levels were significantly lower in OA subjects compared to controls for PPT_TK_Pa (*p* = 0.028), PPT_CK_Pa (*p* = 0.036), and PPT_TK_M (*p* = 0.036) (Table 2). There were no significant differences (*p* ranging from 0.320 to 0.910) between PPT measurements at the patella of target knee (PPT_TK_Pa) and contralateral knee (PPT_CK_Pa), or of averages over the whole target/contralateral knees (PPT_TK, PPT_CK), radius/malleolus (PPT_TK_M, QST_TK_R), suggesting evidence of central sensitization in both mild and advanced OA groups.

The MOAKS was used to assess structural damage in the mild and advanced OA groups (Figure 1). The inter-rater reliability of MRI-assessed MOAKS showed moderate but significant agreement, with an average Kendall’s tau-*b* of 0.51 (range 0.39–0.66) and average Spearman correlation of 0.59 (range 0.48–0.71), demonstrating good agreement between the two readers (see Supplementary Table 1). For measures of MOAKS evaluated structural knee damage, there were significant differences between the advanced and mild OA groups, with statistically significant higher structural damage in the advanced OA group compared with the mild OA group. This included numbers of regions of BMLs (nBML), cartilage degradation (nCD), nOst, in addition to the overall load scores for BML_Load, CD_Load, and Ost_Load (Figure 1C). The severity of effusion Syn was significantly higher in advanced compared with mild OA (*p* < 0.05), but no significant difference in Hoffa’s or total Syn.

**FIGURE 2:**
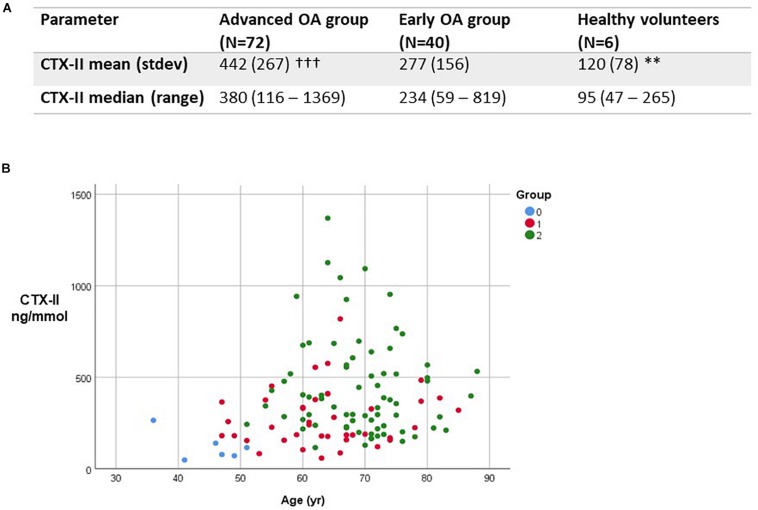
Type II collagen degradation product expression in study. **(A)** Comparison between urinary CTX-II levels in study groups in ng/mmol. ^∗∗^*p* < 0.01, between healthy controls and all OA patients; ^†††^*p* < 0.001 between advanced and early OA groups. **(B)** Variation in levels of CTX-II by subject group and age. Group 0, healthy controls; Group 1, mild OA; Group 2, advanced OA.

We measured urinary type II collagen degradation products (CTX-II) by ELISA in all three study groups (Figure 2). For CTX-II values, the minimum value was 120 ng/mmol, with the maximum 1808 ng/mmol. The range was 1688 ng/mmol. The mean CTX-II value was 450.2 ng/mmol with a *SD* of 302.2. The coefficient of variation across replicate measurements was 67%. Levels of CTX-II were significantly higher in advanced OA in comparison to mild OA (*p* < 0.001). Urinary CTX-II levels were significantly higher in mild and advanced OA compared with healthy controls (*p* < 0.01). We found that urinary CTX-II levels increased with worsening severity of OA and increased with age (Figure 2).

**FIGURE 3:**
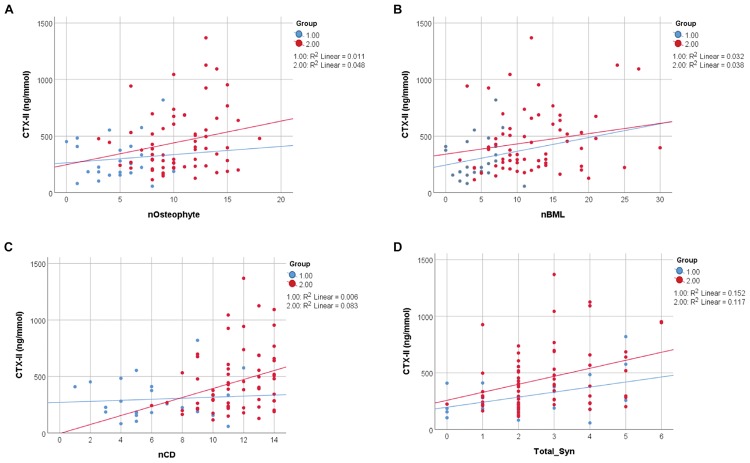
Relation of CTX-II to MRI-defined structural damage in knee OA. Pearson correlation of CTX-II levels with **(A)** the number of osteophytes (nOsteophyte) (*p* = 0.003), **(B)** the number of bone marrow lesions (nBMLs) (*p* = 0.005), **(C)** the number of regions of cartilage degradation (nCD) (*p* = 0.002) and with **(D)** the total synovitis total_Syn (*p* = 0.001). Group 1, mild OA group; Group 2, advanced OA group; Syn, synovitis; BML, bone marrow lesion; CD, cartilage degradation; Ost, osteophyte.

**FIGURE 4:**
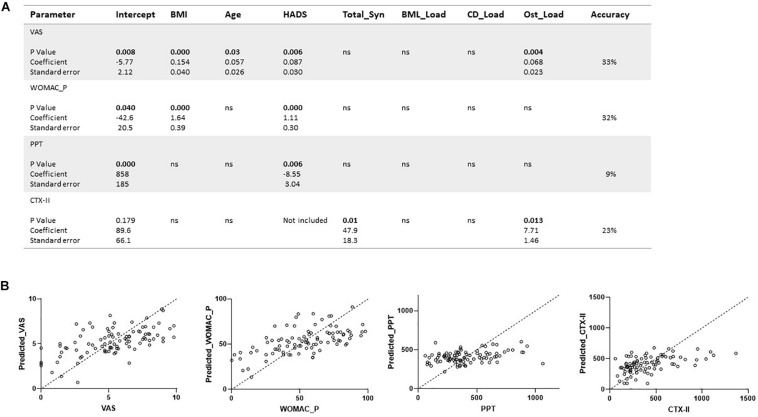
Regression analyses of clinical, biochemical, and structural parameters in knee OA. **(A,B)** Multivariable regression analyses were conducted for continuous variables including VAS, WOMAC pain, urinary CTX-II, MOAKS-defined damage including bone marrow lesion load (BML_Load), total synovitis (total_Syn), cartilage degradation load (CD_Load), osteophyte load (Ost_Load), pain pressure thresholds (PPT), and Hospital Anxiety and Depression Scale (HADS). Regression analysis is shown for the urinary type II collagen degradation product biomarker (CTX-II) in relation to structural damage by osteophytes and synovitis. Covariates were BMI and age in the analysis. Analyses are presented with *p*-values, coefficients, and standard error. There were 78 degrees of freedom.

Bivariate correlation analysis showed significant correlations between most of the measured parameters (Supplementary Tables 1, 2). In particular there were strong correlations between the different pain scores (VAS, WOMAC_P, PPT) and between the pain scores and BMI, age, and HADS, as well as between VAS and WOMAC_P and MRI scores of damage (Supplementary Table 2). Therefore, BMI, age, and HADS were used as covariates and included in the multivariate analysis to assess how pain related to structural damage. Since CTX-II was significantly correlated with BMI and almost significantly correlated with age (Supplementary Table 2), BMI and age were used as covariates and included in the multivariate analysis to assess how CTX-II related to structural damage. The direct correlation of CTX-II to structural damage determined by MOAKS scores for numbers of Ost, BMLs, CD, and total Syn is shown in Figure 3 for mild and advanced OA (Figure 3), with the strongest correlations shown for total Syn. When evaluating the whole dataset of mild and advanced OA combined with BMI and age as covariates, we still found a significant correlation between CTX-II levels and total Syn (total_Syn) (*R* = 0.313, *p* = 0.03), number of Ost (*R* = 0.330, *p* = 0.002), number of BML (*R* = 0.252, *p* = 0.019), and number of regions of CD (*R* = 0.218, *p* = 0.042). We also found CTX-II levels correlated with lesion load scores for CD (*R* = 0.277, *p* = 0.009) and BML (*R* = 0.308, *p* = 0.004). A multivariate analysis using automatic linear modeling determined that the most significant parameters that determined the CTX-II levels were Ost load and total Syn (*p* < 0.05) with a predictive accuracy of 23% (Figure 4).

Multivariate analysis for the pain scores as dependent variables showed that HADS was a highly significant factor (*p* = 0.006) in determining pain for VAS, WOMAC, and PPT, and BMI a very significant factor (*P* < 0.001) for VAS and WOMAC (Figure 4). Only for VAS were there significant predictive effects related to age and to MRI via the Ost load. However, the predictive accuracy was only moderate for VAS and WOMAC at 33% and 32%, respectively, and very low at 9% for PPT.

## Discussion

Knee OA is a multifactorial condition which produces pain and exhibits damage to cartilage, bone, and the development of Syn. Biomarkers are urgently needed to aid patient stratification and for developing improved treatment strategies. We investigated how CTX-II generated during OA, relates to MRI features of knee damage, and showed the most significant relationships were to Syn and Ost. In our study we have shown that pain sensitization was found in mild and advanced OA subjects. We also found PPT measures were not strongly related to structural damage (Figure 4). The strongest associations of WOMAC and VAS reported pain were to HADS and BMI, indicating the difficulty of assigning pain directly to measures of structural damage when there are psychological and physiological confounds in the individual’s perception of pain, and difficulty in deriving causal relationships. We have shown, to our knowledge for the first time, that by combining biomarkers derived from damaged tissue, including type II collagen degradation products and MRI-detected Syn, BMLs, and CD, they can be used to track progression in painful knee OA. The biomarkers we propose could also be used to assess response to future therapeutic interventions including DMOADS.

We found that patient reported pain by WOMAC_P was significantly elevated in people with advanced and mild OA. Pain in OA is thought to arise from richly innervated structures including the synovium and bone, with more recent work reporting expression of neurotrophic factors such as nerve growth factor (NGF) at the osteochondral junction (Sofat et al., 2011). A number of studies have suggested that pain sensitization in OA is mediated by neurotrophic factors expressed within several joint compartments, including the synovium and osteochondral junction (reviewed in Sofat et al., 2011; Neogi et al., 2015; Kuttapitiya et al., 2017). With respect to structural damage assessed by MOAKS, in direct correlations we found that the number of affected regions, rather than the estimated lesion load, most strongly correlate with WOMAC pain (Supplementary Table 2), suggesting it is the development of multiple damage sites that relate to increased pain levels rather than the severity of damage at these sites. However, a multivariate analysis indicated that BMI and HADS were the most significant predictors of pain scores (Figure 4), indicating the complexity of assigning pain directly to measures of structural damage. Overall knee PPT measures were not significantly different between advanced and early OA, and not directly related to structural damage. Neogi et al. (2015) have previously shown that pain sensitization is a significant feature in OA and is related to Syn but not BMLs. Our data contribute further to observations of pain sensitization in OA by demonstrating that sensitization is also detected in early OA disease and could reflect a feature of a threshold of joint damage which is required for sensitization features to develop. We also found that pain sensitization measured by PPT was significantly related to anxiety and depression scores, suggesting a strong psychological component to pain in knee OA, as suggested by other studies (Arendt-Nielsen, 2017; Deveza et al., 2017; O’Neill et al., 2018). Our data show that subjects with advanced OA were older than people with early OA and healthy controls. The advanced OA group had more severe disease as evidenced by greater structural damage by MRI and higher pain scores. With advancing age, OA severity tends to increase, as does BMI, which we also observed.

We found that urinary CTX-II degradation products are strongly related to structural damage and particularly to measures of Ost and Syn. The synovium is a richly innervated part of the joint and has been proposed as a major mediator of pain in OA (Hunter et al., 2011a; Neogi et al., 2015). CTX-II is complementary to pain scores and has potential as a surrogate marker (e.g., in the absence of MRI) of overall damage to aid patient stratification for therapy. Since Syn and Ost are known features of tissue damage in OA, our findings suggest that structures including synovium and bone may be a source of enzymes such as matrix metalloproteinases which are implicated in type II collagen degradation by our group (Kuttapitiya et al., 2017) and others (Nielsen et al., 2008) and not just cartilage as was traditionally described.

The importance of type II collagen degradation has been demonstrated in animal models. In a rat model of OA using anterior cruciate ligament (ACLT) resected rats, Nielsen et al. (2008) showed that protein extracts and histology demonstrated C-terminal telopeptide of type II collagen in protein extracts and histology that was greater than sham-operated rats. CTX-II epitopes were also detected in ACLT resected rat joint sections (Nielsen et al., 2008). In human studies, samples analyzed from the OA initiative (OAI) showed that higher levels of urinary CTX-II were correlated with patellar damage by MRI (*R* = 0.19, *p* = 0.04) (Joseph et al., 2018). A recent analysis evaluated if urinary C-terminal telopeptide of type II collagen (CTX-II) levels are different between people with OA and healthy non-OA controls (Huang et al., 2017). In a meta-analysis, Huang et al. (2017) found CTX-II levels were higher in subjects with OA than in controls. A subgroup analysis showed that CTX-II levels rose with increasing OA severity, suggesting that type II degradation products of type II collagen may be a promising biomarker for OA. Companies have developed ELISAs for Type II collagen degradation specific neoepitopes; Bay-Jensen et al. (2011) developed ELISAs measuring CIIM in synovial fluid/serum, with both compartments showing higher CIIM in OA compared with non-OA samples (*p* < 0.05). Other groups have also reported the validity of using CTX-II degradation products as a biomarker for OA (Van Spil et al., 2015). Data from the CHECK cohort showed that urinary CTX-II levels showed positive associations with Ost area and negative associations with minimum joint space width (Van Spil et al., 2015).

Our study demonstrates that the burden of increasing structural damage in different compartments of the joint, i.e., cartilage, bone, and synovium, are all strongly linked to each other as well as to measures of clinical pain reporting (VAS and WOMAC_P) but *not* to the evoked pain evaluated by pain sensitization measures (Figure 4 and Supplementary Table 2), suggesting PPT is an important independent parameter for characterizing OA subjects. Many reports have investigated structural damage parameters, including BMLs and Syn, independently of each other (Kornaat et al., 2007; Dam et al., 2009; Collins et al., 2016; Everhart et al., 2019). Analysis from the OAI has demonstrated that cartilage defects are independent risk factors for joint replacement in knee OA over a 9-year observation period (Everhart et al., 2019), supporting findings from our study that greater cartilage defects are associated with more symptomatic pain requiring treatment (Figure 1). We also found that greater levels of MOAKS’ derived Syn is associated with higher levels of reported pain, as suggested by other studies (Collins et al., 2016). In a nested case–control study conducted as part of the Foundation for the National Institutes of Health Biomarkers Consortium Project (FNIH), cartilage thickness, cartilage morphology, effusion Syn/Hoffa Syn, and meniscal pathology were associated with OA progression over 2 years using a multivariable logistic regression model (Collins et al., 2016), suggesting that biomarkers which incorporate structural changes including CD and Syn in relation to symptom progression, as we found in our study, could be very useful markers in future studies of novel therapeutic agents. Other studies have supported our finding that type II collagen degradation and MRI-detected tissue damage can be used to compare the diagnostic and predictive abilities of different combinations of imaging and biochemical markers to track OA progression and severity (Collins et al., 2016). However, our study found greater correlation between distinct structural damage parameters including BMLs, CD, Ost load, and Syn than previous cohort studies. The reason for the differences found may be that we used a cross-sectional study design in comparison to other studies of longitudinal cohorts (Van Spil et al., 2015; Joseph et al., 2018), thereby finding greater correlation with a broader range of damage features than the distinct changes in structures found in longitudinal studies. More recently, a machine learning algorithm has been used to combine data from the FNIH Biomarkers Consortium to show that MRI-based structural damage measures were better predictors of OA progression than biochemical markers (Nielsen et al., 2008). In contrast, baseline variables that contributed to progression included BMLs, Ost, medial meniscal extrusion, and urine C-terminal cross-linked type II collagen telopeptide (Nelson et al., 2019).

Limitations of our study include the fact that data at one time point only were evaluated in our cross-sectional analysis. The potential of confounding factors for age and BMI in introducing bias in our study results were also a consideration; therefore, data were corrected for covariates including age and BMI where possible. Bias can occur when recruiting subjects into clinical studies, especially since we had a wide age range for recruitment into the study. We aimed to reduce bias as much as possible by recruiting consecutive subjects who were referred to our clinical services consecutively, provided they gave consent. For healthy controls, they were recruited in an unbiased manner as much as possible through adverts placed in clinic areas. To follow-on from our cross-sectional study, future longitudinal studies of our cohort will be needed to evaluate long-term outcomes for pain, function, and biomarkers. Our analysis will also be aided by future interrogation of wet biomarkers in particular in larger longitudinal cohort studies. Although we determined significant correlations between pain measures and the fluid marker CTX-II, with measures of structural damage (Figure 4), the accuracy was low suggesting there may be other factors determining pain that we have not taken into account (e.g., MRI structural damage scores were of the most painful knee, but pain and CTX-II measures are whole body related). A limited number of healthy controls were studied to obtain indicative parameterization of CTX-II and PPT scores in disease-free volunteers at an age close to early OA. Further studies with a larger control cohort across the full age range are still needed to fully determine threshold levels related to disease, as in this study our primary aim was to evaluate relationships between structural, fluid biomarkers, and pain parameters within the OA groups.

## Conclusion

In summary, data from our study demonstrate how people with both mild and advanced OA have features of pain sensitization, which is likely triggered by tissue damage in OA in several compartments, including bone, cartilage, and synovium. The MRI and urinary CTX-II biochemical biomarkers that we identified have potential use in the clinic to assess and monitor response to treatments in painful knee OA, a prevalent condition in which robust biomarkers are needed to assess disease progression and guide treatments.

## Data Availability Statement

The datasets generated for this study are available on request to the corresponding author.

## Ethics Statement

The studies involving human participants were reviewed and approved by the London Surrey Borders Ethics Committee. The patients/participants provided their written informed consent to participate in this study.

## Author Contributions

NS wrote the study protocol and associated documents, coordinated the implementation of the study, collated and managed the study data, conducted data analysis, and drafted the manuscript. VE and CH interpreted the knee MRI scores for data analysis. AK, LA, SK, AH, GW, and FH supported NS in the study design, data collection, and study analysis.

## Conflict of Interest

The authors declare that the research was conducted in the absence of any commercial or financial relationships that could be construed as a potential conflict of interest.
