# Supplementary material for: Biomarkers in Painful Symptomatic Knee OA Demonstrate That MRI Assessed Joint Damage and Type II Collagen Degradation Products Are Linked to Disease Progression
Source: Front Neurosci. 2019 Oct 15;13:1016. doi: 10.3389/fnins.2019.01016 (PMC6803383; doi:10.3389/fnins.2019.01016)
Supplement: Supplementary file 1 [file Table_1.DOCX]

Supplementary Table 1

| **Parameter** | **N** | **Kendall's tau-b** | **Spearman** | **p** |  |
| --- | --- | --- | --- | --- | --- |
| BML_N | 1096 | 0.557 | 0.613 | 0.000 |  |
| BML_size | 1096 | 0.661 | 0.709 | 0.000 |  |
| BML_%Vol | 1096 | 0.434 | 0.478 | 0.000 |  |
| BML_N_total | 73 | 0.527 | 0.681 | 0.000 |  |
| CD_size | 1096 | 0.528 | 0.598 | 0.000 |  |
| CD_%FT | 1096 | 0.557 | 0.613 | 0.000 |  |
| CD_N_total | 73 | 0.457 | 0.564 | 0.000 |  |
| Ost_Size | 1096 | 0.504 | 0.572 | 0.000 |  |
| Ost_N_total | 73 | 0.387 | 0.49 | 0.000 |  |

**Supplementary Table 1**. Inter-rater evaluations made over: A) N=73 patients for the total numbers of lesion regions for BML and CD (BML_N_total, CD_N_total) and total number of osteophytes (Ost_N_total) per patient; B) N=1096 total anatomical regions assessed for lesion size (BML_size, CD_size, Ost_size), the percentage of BML lesion volume (BML_%vol) and CD lesion full thickness (CD_%FT), and osteophyte size (Ost_size). Parameters are as defined by Hunter *et al.* (13) for which size and % parameters have a range 0 to 3.

Supplementary Table 2

| **Correlations between dependent variables and clinical and MRI scores** | | | | | | | | | | | | | | | |
| --- | --- | --- | --- | --- | --- | --- | --- | --- | --- | --- | --- | --- | --- | --- | --- |
|  | | **VAS** | **WOMAC_P** | **PPT** | **CTX-II** | **BMI** | **Age** | **HADS** | **Total_Syn** | **BML_Load** | **CD_Load** | **Ost_Load** | **nBML** | **nCD** | **nOst** |
| **VAS** | *R* |  | **.757^**^** | **-.366^**^** | **.194^*^** | **.415^**^** | **.288^**^** | **.282^**^** | 0.103 | **.205^*^** | **.323^**^** | **.379^**^** | **.329^**^** | **.330^**^** | **.431^**^** |
|  | *p* |  | **0.000** | **0.000** | **0.036** | **0.000** | **0.001** | **0.001** | 0.328 | **0.048** | **0.002** | **0.000** | **0.001** | **0.001** | **0.000** |
|  | *N* |  | 126 | 124 | 118 | 125 | 126 | 126 | 93 | 93 | 93 | 93 | 93 | 93 | 93 |
| **WOMAC_P** | *R* | **.757^**^** |  | **-.369^**^** | **.221^*^** | **.483^**^** | **.224^*^** | **.408^**^** | 0.178 | 0.060 | 0.199 | **.259^*^** | 0.193 | **.376^**^** | **.364^**^** |
|  | *p* | **0.000** |  | **0.000** | **0.016** | **0.000** | **0.012** | **0.000** | 0.089 | 0.566 | 0.056 | **0.012** | 0.064 | **0.000** | **0.000** |
|  | *N* | 126 |  | 124 | 118 | 125 | 126 | 126 | 93 | 93 | 93 | 93 | 93 | 93 | 93 |
| **PPT** | *R* | **-.366^**^** | **-.369^**^** |  | -0.021 | **-.314^**^** | -0.131 | **-.324^**^** | -0.020 | -0.069 | -0.082 | -0.163 | -0.114 | -0.157 | -0.112 |
|  | *p* | **0.000** | **0.000** |  | 0.818 | **0.000** | 0.147 | **0.000** | 0.849 | 0.516 | 0.437 | 0.120 | 0.280 | 0.136 | 0.288 |
|  | *N* | 124 | 124 |  | 117 | 123 | 124 | 124 | 92 | 92 | 92 | 92 | 92 | 92 | 92 |
| **CTX-II** | *R* | **.194^*^** | **.221^*^** | -0.021 |  | **.260^**^** | 0.172 | 0.107 | **.363^**^** | **.346^**^** | **.348^**^** | **.398^**^** | **.295^**^** | **.331^**^** | **.313^**^** |
|  | *p* | **0.036** | **0.016** | 0.818 |  | **0.005** | 0.063 | 0.249 | **0.001** | **0.001** | **0.001** | **0.000** | **0.005** | **0.002** | **0.003** |
|  | *N* | 118 | 118 | 117 |  | 117 | 118 | 118 | 88 | 88 | 88 | 88 | 88 | 88 | 88 |

**Supplementary Table 2**. Pearson correlation *R* between dependent variables of the pain scores and clinical and MRI variables. *p* is the two-tailed significance, N the total numbers of patients used in each correlation.
